# Varied Growth Response of Cogongrass Ecotypes to Elevated CO_2_

**DOI:** 10.3389/fpls.2015.01182

**Published:** 2016-01-05

**Authors:** G. Brett Runion, Stephen A. Prior, Ludovic J. A. Capo-chichi, H. Allen Torbert, Edzard van Santen

**Affiliations:** ^1^National Soil Dynamics Laboratory, Agricultural Research Service, United States Department of AgricultureAuburn, AL, USA; ^2^Alberta Innovates Technology FuturesVegreville, AB, Canada; ^3^Department of Crop, Soil and Environmental Sciences, Auburn UniversityAuburn, AL, USA

**Keywords:** carbon dioxide, global change, *Imperata cylindrica*, invasive weed, nitrogen use efficiency, water use efficiency

## Abstract

Cogongrass [*Imperata cylindrica* (L.) P. Beauv] is an invasive C_4_ perennial grass which is listed as one of the top ten worst weeds in the world and is a major problem in the Southeast US. Five cogongrass ecotypes [Florida (FL), Hybrid (HY), Louisiana (LA), Mobile (MB), and North Alabama (NA)] collected across the Southeast and a red-tip (RT) ornamental variety were container grown for 6 months in open top chambers under ambient and elevated (ambient plus 200 ppm) atmospheric CO_2_. Elevated CO_2_ increased average dry weight (13%) which is typical for grasses. Elevated CO_2_ increased height growth and both nitrogen and water use efficiencies, but lowered tissue nitrogen concentration; again, these are typical plant responses to elevated CO_2_. The HY ecotype tended to exhibit the greatest growth (followed by LA, NA, and FL ecotypes) whiles the RT and MB ecotypes were smallest. Interactions of CO_2_ with ecotype generally showed that the HY, LA, FL, and/or NA ecotypes showed a positive response to CO_2_ while the MB and RT ecotypes did not. Cogongrass is a problematic invasive weed in the southeastern U.S. and some ecotypes may become more so as atmospheric CO_2_ continues to rise.

## Introduction

Invasive plants are estimated to cost U.S. agricultural and forest producers 34 billion dollars annually from decreased productivity and increased cost of weed control and are considered to be a major threat to the Earth’s biodiversity (Pimentel, 2002). Elevated CO_2_ stimulates plant photosynthesis, resource use efficiency, and biomass production (Amthor, 1995) which may affect the physiology and competitiveness of invasive plants. However, the effects of elevated CO_2_ on invasive plants remains an understudied aspect of global change research. Bright (1998) summarizes, “Fast-growing, highly invasive plants may also be able to profit directly from the atmosphere’s increased carbon content...any slower-growing natives would tend to lose out to the invaders.” It has been suggested that the increase in the atmospheric concentration of CO_2_ since the beginning of the 20th century may be a primary factor affecting the establishment and spread of some invasive species (Ziska, 2003).

Invasive plants can disrupt terrestrial ecosystems, particularly in the southeastern U.S. with its numerous ports of entry and mild climate. One example that has become a serious problem is cogongrass [*Imperata cylindrica* (L.) P. Beauv], a perennial grass native to Southeast Asia which was introduced into the southeastern U.S. in the early 1900s (Tabor, 1949) for forage, erosion control and as packing material (Bryson and Carter, 1993). It is a widespread invader to warmer regions (>500 million ha worldwide), is tolerant of shade, poor soils, and drought and naturalizes aggressively in dense monocultures which displace native plants (Bryson and Carter, 1993). Cogongrass is one of the top ten worst weeds in the world (Holm et al., 1991) and is a Federal Noxious Weed (Miller et al., 2010). Cogongrass is a major problem in the Southeast on disturbed lands such as forest plantations and roadsides and may become problematic on agricultural lands (Patterson et al., 1980). It is present in five or more varieties including a commercially available red-tip (RT; ‘Red Baron’) ornamental sold by nurseries in some states (Capo-chichi et al., 2008). Sale of this RT variety is prohibited in some southern states and removal of prior plantings has been recommended since it has viable pollen that might spread to invasive cogongrass plants and has been known to revert back to the green aggressive type (Miller et al., 2010).

It has been suggested that populations introduced from several origins over an extended period of time should have higher genetic diversity than populations that were introduced only a few times or from a single source (Pappert et al., 2000). For example, cogongrass was first introduced to Alabama from Japan (Tabor, 1952), but has likely also arrived from other locations to different ports of entry. Genetic characterization of differing populations of cogongrass may help explain spread dynamics and means of establishment (Capo-chichi et al., 2008). These investigators determined that genetic variation within and between cogongrass sites in the southern U.S. was quite large given how recently it was introduced. Spread dynamics were found to be greatly influenced by anthropogenic activities (e.g., soil disturbance, canopy removal) compared to natural factors which may accelerate opportunities for bringing together cross-compatible species previously isolated by ecology and/or geography. The objective of this study was to evaluate the response of five cogongrass ecotypes collected across the southeastern U.S. plus the RT variety to ambient and elevated atmospheric CO_2_. This is the first study to examine the effects of elevated CO_2_ on cogongrass and the first for any weed species to look at potential differences among ecotypes.

## Materials and Methods

The study was conducted at the soil bin facilities at the USDA-ARS National Soil Dynamics Laboratory, Auburn, Alabama. The bin used for the experimental setup is 6 m wide and 76 m long and has been modified for container studies; modifications consisted of installing a geomembrane liner (20 mL) and gravel drain system to ensure a good working surface and drainage for container studies. Open top field chambers (OTC; Rogers et al., 1983a), encompassing 7.3 m×7.3 m of ground surface area, were used to continuously deliver target CO_2_ concentrations of ambient or ambient plus 200 μmol mol^-1^ (elevated) using a delivery and monitoring system described by Mitchell et al. (1995).

The six cogongrass ecotypes used in this study were from a collection maintained at Auburn University and included Louisiana, North Alabama, Florida, Mobile, a LA-MB HY, and RT. The MB ecotype was from the suspected point of introduction near Grand Bay in Mobile County, AL as described by Tabor (1952). The RT represents a commercially available variety that can be found in ornamental nurseries. Rhizomes were collected, stored in plastic bags, and transported to glasshouses at the Plant Sciences Research Center of the Alabama Agricultural Experiment Station on the campus of Auburn University for molecular analysis. An out-crossing involving the non-native and native species led to different genotypes such as the LA-MB HY. Amplified Fragment Length Polymorphism (AFLP) confirmed that the cogongrass ecotype LA-MB HY was derived from non-native (wild type cogongrass ecotype) and native (non-invasive cogongrass ecotype) species (Capo-chichi et al., 2008). The atpB-rbcL non-coding spacer of chloroplast DNA revealed that only a few nucleotide substitutions contributed to the variation among the wild cogongrass MB ecotype and the RT (data not shown).

Plants were grown in a peat-based general purpose growing medium (PRO-MIX Bx, Premier Horticulture Inc., Quakertown, PA 18951, USA) in 1.65 L tree-pots (Short One Tree-pot, 10 cm × 23 cm, Stuewe and Sons Inc., Corvallis, OR 97333, USA) in a glasshouse for establishment (∼3 wk). Plants were then transplanted into 10.65 L tree pots (TPOT4 Round Tree-pot, 22 cm × 39 cm, Stuewe and Sons Inc., Corvallis, OR 97333, USA) containing the same standard growth medium described above. Forty-eight containers of each ecotype were selected for use in the study. These plants were ranked, according to size and placed into four groups of 12 containers each, representing the largest 12 first in declining order down to the smallest 12; one container from each group was randomly assigned to each of the 12 OTCs (i.e., four containers of each plant ecotypes in each chamber). The study was conducted as a randomized complete block design with the six blocks occurring along the length of the soil bin. Plants were fertilized monthly with Miracle-Gro (15:30:15, N:P:K; Scotts Products Inc., Marysville, OH, USA) according to manufacture recommendations by mixing 600 g Miracle-Gro in 130 L deionized water; each plant received 500 mL of this solution. Containers were subjected to ambient rainfall and watered once or twice a week (1 L per container) to prevent drought-induced plant mortality.

Prior to harvest, WUE was calculated from LI-6400 Portable Photosynthesis System (LI-COR, Inc., Lincoln, NE, USA) measurements. After 6 months, height was measured and the aboveground portions were harvested by severing the plant at the ground-line. Roots were separated from the growing medium using the sieve method (Bohm, 1979). Above- and belowground plant components were then dried separately in a forced-air oven at 55°C to a constant weight, and dry weights recorded. Subsamples of above- and belowground biomass (1 mm sieve) were analyzed separately for N by dry combustion using a LECO TruSpec analyzer (LECO Corp., St. Joseph, MI, USA). Prior to analyses, data from the four containers of each ecotype within each chamber were averaged making the chamber the experimental unit (*N* = 6). Statistical analyses were conducted using the Mixed Models procedure (Proc Mixed) from SAS (Littell et al., 1996). In all cases, differences were considered significant at *P* ≤ 0.05 and trends were recognized at 0.05 > *P* ≤ 0.10.

## Results

**FIGURE 1:**
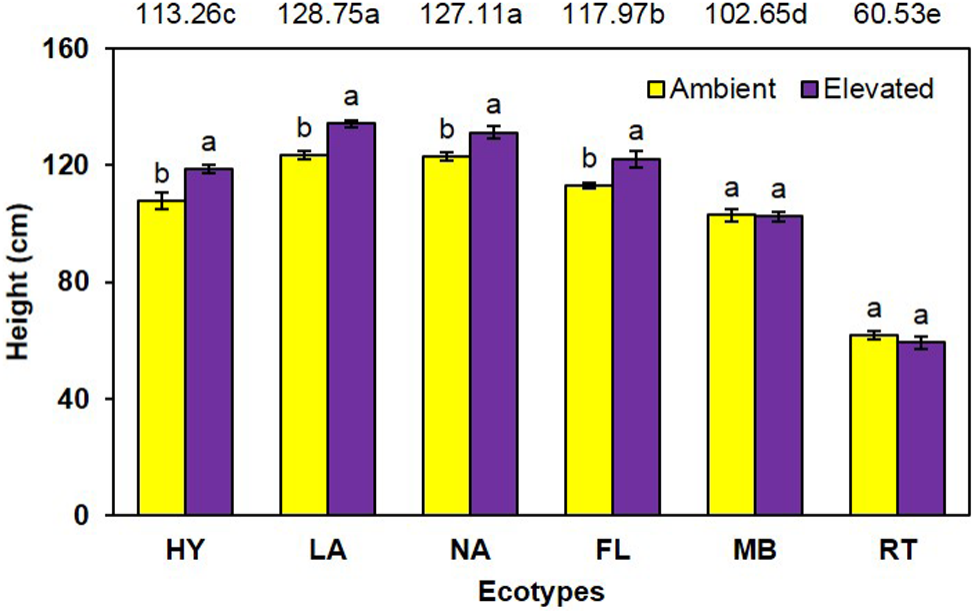
**Height of cogongrass ecotypes under ambient and elevated atmospheric CO_2_ (HY, Hybrid; LA, Louisiana; NA, North Alabama; FL, Florida; MB, Mobile; RT, Red-tip)**. *N* = 6. Means with standard errors are shown; standard errors reflect the variability in the data and are not a means separation technique. Main effects of CO_2_ (*P* < 0.001), ecotype (*P* < 0.001), and their interaction (*P* = 0.001) were significant. Bars with different letters show a significant effect of CO_2_ for each ecotype; main effect ecotype means shown at the top of the graph (means followed by the same letter are not significantly different).

Cogongrass height was significantly increased (*P* < 0.001) under elevated atmospheric CO_2_ (111.3 cm) compared to ambient conditions (105.4 cm) when averaged across all ecotypes (**Figure 1**). When averaged across CO_2_ concentrations, significant differences in height (*P* < 0.001) were noted among the ecotypes (i.e., LA = NA > FL > HY > MB > RT). Further, a significant interaction (*P* = 0.001) showed that height was increased by elevated CO_2_ for LA, NA, FL, and HY only (**Figure 1**).

**FIGURE 2:**
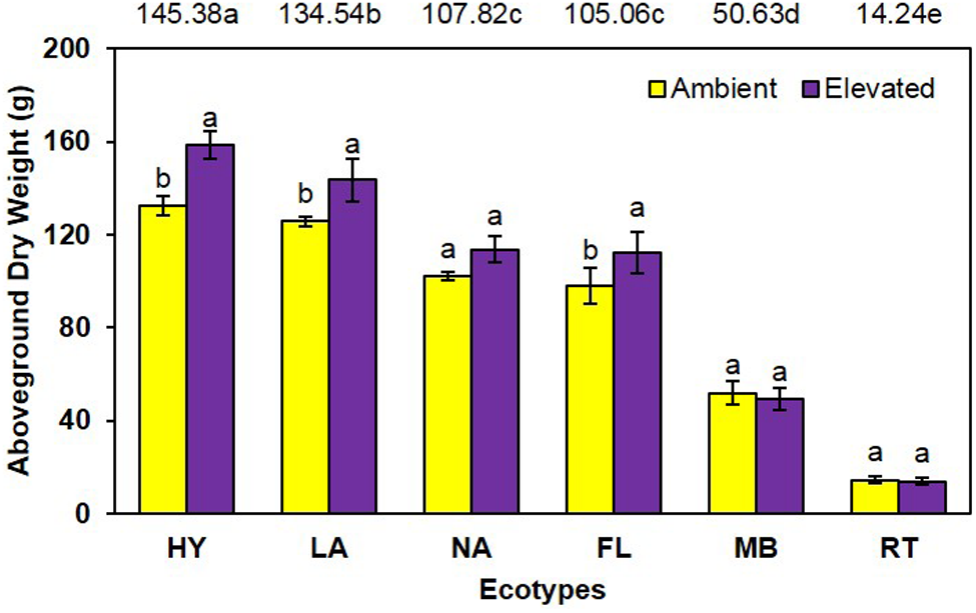
**Aboveground biomass of cogongrass ecotypes under ambient and elevated atmospheric CO_2_ (HY, Hybrid; LA, Louisiana; NA, North Alabama; FL, Florida; MB, Mobile; RT, Red-tip)**. *N* = 6. Means with standard errors are shown; standard errors reflect the variability in the data and are not a means separation technique. Main effects of CO_2_ (*P* = 0.001) and ecotype (*P* < 0.001) were significant and their interaction showed a trend (*P* = 0.095). Bars with different letters show a significant effect of CO_2_ for each ecotype; main effect ecotype means shown at the top of the graph (means followed by the same letter are not significantly different).

Similarly, aboveground dry weight was significantly increased (*P* = 0.001) 13% under elevated (98.5 g) compared with ambient (87.4 g) CO_2_ (**Figure 2**). A significant main effect of ecotype (*P* < 0.001) was also observed (i.e., HY > LA > NA = FL > MB > RT) as was a trend for an interaction (*P* = 0.095), where dry weight was increased by elevated CO_2_ for HY, LA, and FL only (**Figure 2**).

**FIGURE 3:**
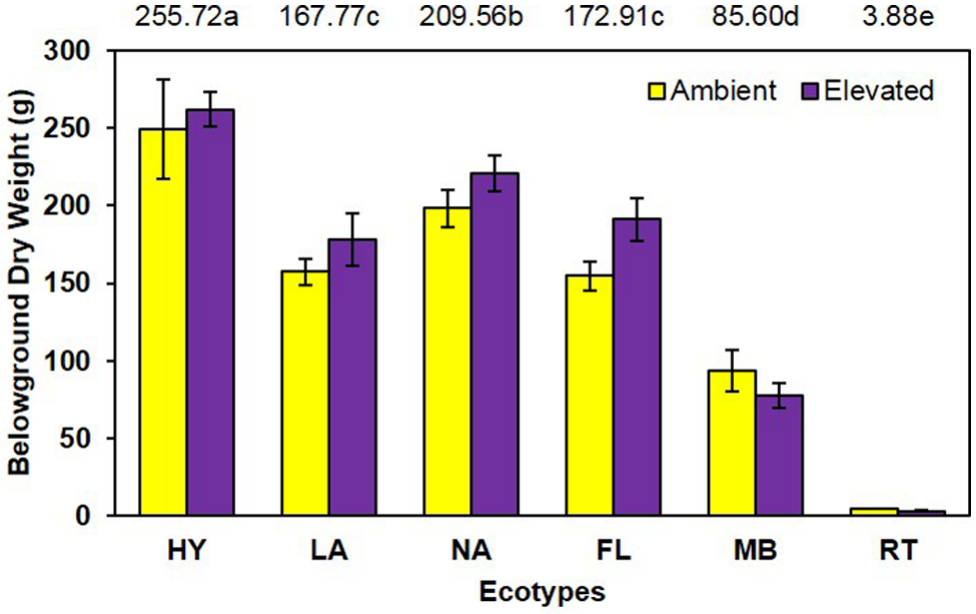
**Belowground biomass of cogongrass ecotypes under ambient and elevated atmospheric CO_2_ (HY, Hybrid; LA, Louisiana; NA, North Alabama; FL, Florida; MB, Mobile; RT, Red-tip)**. *N* = 6. Means with standard errors are shown; standard errors reflect the variability in the data and are not a means separation technique. Main effect of ecotype (*P* < 0.001) was significant while the main effect of CO_2_ showed a trend (*P* = 0.118) and their interaction (*P* = 0.487) was not significant. The omission of letters above bars indicates no effect of CO_2_ for any ecotype; main effect ecotype means shown at the top of the graph (means followed by the same letter are not significantly different).

Although elevated CO_2_ resulted in a slight increase (8.9%) in belowground dry weight (elevated = 155.6 g vs. ambient = 142.9 g; **Figure 3**), this effect was not statistically significant (*P* = 0.118). However, the main effect of ecotype was significant (*P* < 0.001; HY > NA > FL = LA > MB > RT. No significant CO_2_ by ecotype interaction (*P* = 0.487) was noted for this measure.

**FIGURE 4:**
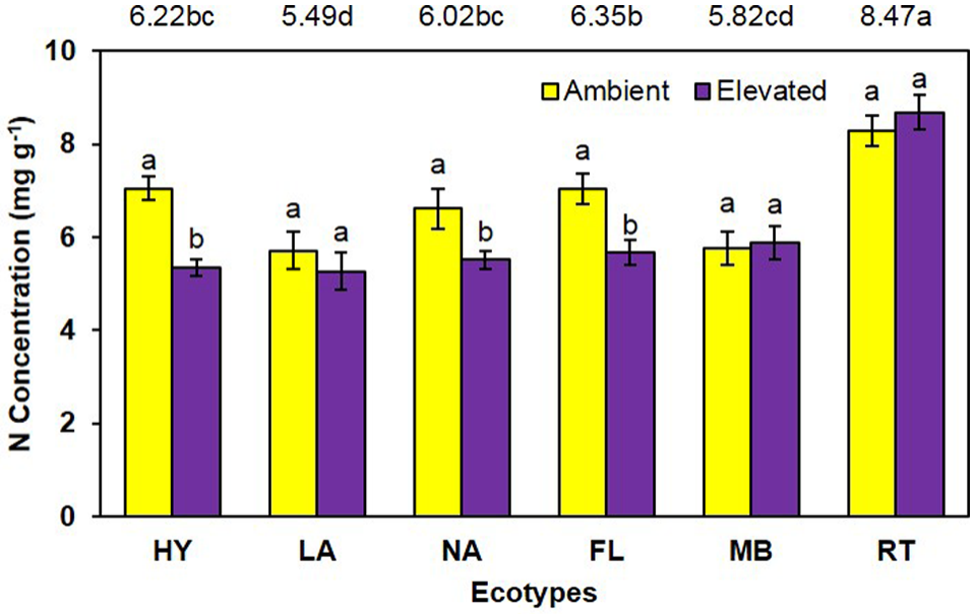
**Total plant nitrogen concentration of cogongrass ecotypes under ambient and elevated CO_2_ (HY, Hybrid; LA, Louisiana; NA, North Alabama; FL, Florida; MB, Mobile; RT, Red-tip)**. *N* = 6. Means with standard errors are shown; standard errors reflect the variability in the data and are not a means separation technique. Main effects of CO_2_ (*P* < 0.001), ecotype (*P* < 0.001), and their interaction (*P* = 0.008) were significant. Bars with different letters show a significant effect of CO_2_ for each ecotype; main effect of ecotype means shown at the top of the graph (means followed by the same letter are not significantly different).

**FIGURE 5:**
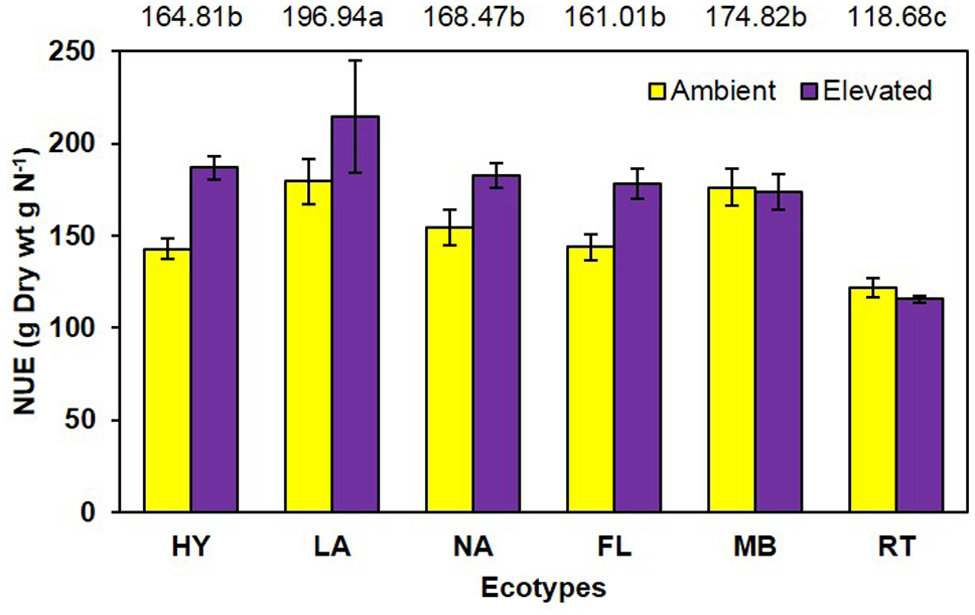
**Nitrogen use efficiency of cogongrass ecotypes under ambient and elevated CO_2_ (HY, Hybrid; LA, Louisiana; NA, North Alabama; FL, Florida; MB, Mobile; RT, Red-tip)**. *N* = 6. Means with standard errors are shown; standard errors reflect the variability in the data and are not a means separation technique. Main effects of CO_2_ (*P* = 0.001) and ecotype (*P* < 0.001) significant; their interaction (*P* = 0.118) showed a trend. The omission of letters above bars indicates no effect of CO_2_ for any ecotype; main effect ecotype means shown at the top of the graph (means followed by the same letter are not significantly different).

Unlike growth measurements, the main effect of CO_2_ showed significantly lower (*P* < 0.001) tissue nitrogen concentration [N] under elevated (6.06 mg N/g) than ambient (6.74 mg N/g) CO_2_ (**Figure 4**). A significant main effect of ecotype (*P* < 0.001) indicated that RT > FL = HY = NA > MB = LA. A significant interaction of CO_2_ with ecotype (*P* = 0.008) showed that [N] was decreased by elevated CO_2_ for the FL, HY, and NA ecotypes only (**Figure 4**). Calculations of NUE as g plant biomass produced per g plant N were significant (*P* = 0.001) for the main effect of CO_2_ (ambient = 153.1 vs. elevated = 175.2; **Figure 5**). Ecotype, averaged across both CO_2_ treatments, significantly (*P* < 0.001) affected NUE (i.e., LA > MB = NA = HY = FL > RT). The CO_2_ by ecotype interaction was not significant for NUE (*P* = 0.118); however, NUE showed a similar response as other variables in that FL, HY, LA, and NA were numerically higher under elevated CO_2_ while MB and RT were actually slightly lower (**Figure 5**).

**FIGURE 6:**
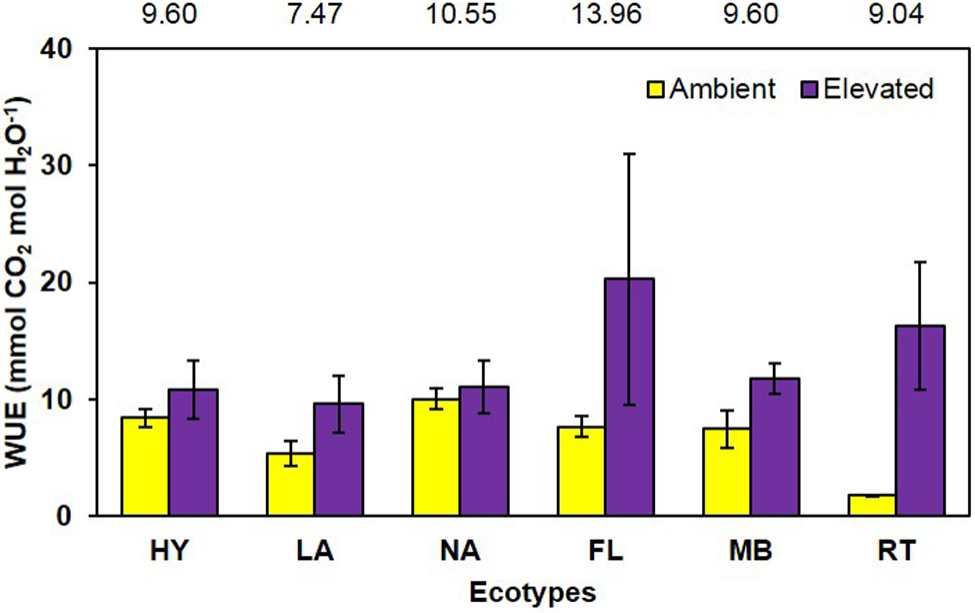
**Water use efficiency of cogongrass ecotypes under ambient and elevated CO_2_ (HY, Hybrid; LA, Louisiana; NA, North Alabama; FL, Florida; MB, Mobile; RT, Red-tip)**. *N* = 6. Means with standard errors are shown; standard errors reflect the variability in the data and are not a means separation technique. Main effect of CO_2_ (*P* = 0.001) was significant while ecotype (*P* = 0.513) and their interaction (*P* = 0.226) were not. The omission of letters above bars indicates no effect of CO_2_ for any ecotype; main effect ecotype means shown at the top of the graph.

Water use efficiency, calculated from LICOR gas exchange measurements as mmol CO_2_ per mol H_2_O, was significantly increased (*P* = 0.001) 96% by growth in elevated CO_2_ (ambient = 6.8 vs. elevated = 13.3; **Figure 6**). The main effect of ecotype (*P* = 0.513) and its interaction with CO_2_ (*P* = 0.226) did not affect WUE (**Figure 6**).

## Discussion

Cogongrass growth parameters were increased when exposed to elevated CO_2_, which is typical of most plants (Rogers et al., 1994; Amthor, 1995). Although height was only slightly higher (5.6%; **Figure 1**), aboveground dry weight increase (12.7%; **Figure 2**) was in a range (10–15%) typical of C_4_ plant response to CO_2_ enrichment (Kimball, 1983; Prior et al., 2003). The fact that responsive ecotypes showed growth responses to elevated CO_2_ typical for C_4_ plants suggests that their invasive potential will not be altered as atmospheric CO_2_ continues to rise, but does indicate that some ecotypes of this serious invasive weed may become more problematic.

Cogongrass ecotype also affected both height and aboveground dry weight; in general, the MB and RT were smaller than the other ecotypes. The significant interactions for these variables further showed that MB and RT were not responsive to CO_2_ concentration, while the other ecotypes tended to be larger under high CO_2_. It is interesting to note that HY tended to exhibit the greatest response to elevated CO_2_ among ecotypes even though it was a cross from the MB ecotype which was not responsive. It is not uncommon for HYs to exhibit growth responses that either differ from or exceed their progenitors (Capo-chichi et al., 2008). This is, after all, why plant breeding programs exist for virtually all important crop species.

Despite the importance of root systems in attaining essential soil resources (i.e., water and nutrients), their response to CO_2_ has received less attention than aboveground parts; however, it has been reported that roots often exhibit a larger response to elevated CO_2_ than other plant organs (Rogers et al., 1994). In contrast, our study showed no increase in root dry weight under elevated CO_2_ and no significant interaction with cogongrass ecotype. It is interesting to note that, despite the lack of significance, the belowground response pattern (**Figure 3**) was similar to that seen for aboveground growth (**Figure 2**). Further, ecotype effect also followed the same general pattern as aboveground dry weight in that MB and RT were smaller than the other ecotypes.

Given that weed species are more likely to have greater genetic diversity and physiological plasticity (compared to crops), they are more likely to be able to adapt to a changing environment (Ziska and Runion, 2007). This may have significant implications for developing effective weed control stategies given that elevated CO_2_ may increase herbicide tolerance in some weeds due to a herbicide dilution effect caused by increased growth, as well as other potential CO_2_-induced changes in plant morphology, biochemistry, and physiology (Ziska et al., 1999; Ziska and Teasdale, 2000; Archambault et al., 2001). However, increased herbicide tolerance under elevated CO_2_ is not always observed (Marble et al., 2015). How cogongrass herbicide efficacy will be impacted by elevated CO_2_ is not known and deserves futher study.

Total plant nitrogen concentration was reduced under elevated CO_2_ (**Figure 4**). As with plant growth, this is a common response to high CO_2_ (Rogers et al., 1994; Norby et al., 2001; Prior et al., 2008; Runion et al., 2009). This is a result of increased plant growth under elevated CO_2_ causing a dilution effect on nutrient concentrations (Rogers et al., 1994, 1999). The RT ecotype had the highest [N] and LA was lowest (**Figure 4**). RT had the smallest growth which likely resulted in the high [N]; it is unclear why MB did not exhibit this pattern given it also had less growth. A significant CO_2_ by ecotype interaction indicated that [N] was lowered by elevated CO_2_ in FL, HY, and NA only. In general, these ecotypes had a larger dilution effect due to greater growth **Figures 1** and **2**); however, why LA did not follow this pattern is not known.

Another common response to elevated CO_2_ is increased NUE (Rogers et al., 1994) as observed in this study (**Figure 5**). Nutrient use efficiency (unit of biomass produced per unit of nutrient) generally increases under elevated CO_2_ as plants are able to produce more biomass with available nutrients. Ecotype also affected NUE with LA being highest and RT lowest. Although the CO_2_ by ecotype interaction was not significant, the response pattern was similar to other variables in that NUE was numerically higher for FL, HY, LA, and NA but not MB and RT under elevated CO_2_.

As with NUE, it is also common for plants grown under elevated CO_2_ to exhibit increases in water use effiency (Rogers and Dahlman, 1993). In general, C_3_ plants exhibit increased photosynthesis and decreased stomatal conductance under elevated CO_2_ (Amthor and Loomis, 1996), leading to increased WUE. However, the CO_2_-concentrating mechanism used by C_4_ species limits their photosynthetic response to CO_2_ enrichment (Amthor and Loomis, 1996), but they do tend to show decreased stomatal conductance which often increases WUE (Rogers et al., 1983b). This was observed in the current study, in that photosynthesis was not significantly affected by CO_2_ concentration (ambient = 2.13, elevated = 2.61 μmol CO_2_ m^-2^ s^-1^; *P* = 0.14), while stomatal conductance tended to be decreased (ambient = 0.018, elevated = 0.014 mol H_2_O m^-2^ s^-1^; *P* = 0.06) under elevated CO_2_ (full data not shown). These responses led to a large increase in WUE (96%) under elevated CO_2_ (**Figure 6**). Ecotype and its interaction with CO_2_ did not affect WUE.

## Conclusion

Cogongrass is one of the top ten worst weeds in the world and is listed as a Federal Noxious Weed. Since its introduction to the Southeastern U.S. it has become a major problem in forest plantations, roadsides, and agricultural systems due to its aggressive ability to develop dense monocultures which can compete with and displace desirable species. This is the first study to examine the effects of elevated CO_2_ on cogongrass and the first for any weed species to look at potential differences among ecotypes. In our study, elevated CO_2_ (averaged across ecotypes) increased height, biomass, and both nitrogen and water use efficiencies, but lowered tissue nitrogen concentration; again, these are typical C_4_ plant responses to elevated CO_2_. In general, the HY ecotype tended to exhibit the greatest growth (followed by LA, NA, and FL) while RT and MB ecotypes were smallest. Interactions of CO_2_ with ecotype showed that MB and RT ecotypes did not respond to CO_2_. This lack of response to CO_2_ for RT (sold commerically as ‘Red Baron’) is significant since concerns over its potential to spread into the native landscape should not be exacerbated by the rising atmospheric CO_2_ concentration. Nevertheless, it is still prohibited for sale in some states and its removal has been recommended. However, HY, LA, FL, and/or NA ecotypes responded positively to elevated CO_2_, suggesting some ecotypes of this serious invasive weed may become more problematic in a future CO_2_-enriched environment. These findings may influence development of future cogongrass control strategies, a subject area requiring further investigation.

## Author Contributions

GB conceived and conducted the research, collected and analyzed the data and co-wrote the manuscript; SP assisted with conducting the research, collecting the data, and co-wrote the manuscript; LC provided the cogongrass ecotypes, co-wrote and reviewed the manuscript; HT assisted with conducting the research and reviewed the manuscript; ES assisted with providing the cogongrass ecotypes and reviewed the manuscript.

## Conflict of Interest Statement

The authors declare that the research was conducted in the absence of any commercial or financial relationships that could be construed as a potential conflict of interest.

## ABBREVIATIONS

FL: Florida cogongrass ecotype
HY: Louisiana/Mobile hybrid cogongrass ecotype
LA: Louisiana cogongrass ecotype
MB: Mobile cogongrass ecotype
NA: North Alabama cogongrass ecotype
NUE: nitrogen use efficiency
OTC: Open Top Chamber
RT: red-tip ornamental cogongrass variety
WUE: water use efficiency

## References

[B1] Amthor J. S. (1995). Terrestrial higher-plant response to increasing atmospheric [CO_2_] in relation to the global carbon cycle. *Global Change Biol.* 1 243–274. 10.1111/j.1365-2486.1995.tb00025.x

[B2] Amthor J. S. Loomis R. S. (1996). “Integrating knowledge of crop responses to elevated CO_2_ and temperature with mechanistic simulation models: model components and research needs,” in *Carbon Dioxide and Terrestrial Ecosystems*, eds Koch G. W. Mooney H. A. (San Diego, CA: Academic Press), 317–346.

[B3] Archambault D. J. Li X. Robinson D. O’Donovan J. T. Klein K. K. (2001). The effects of elevated CO_2_ and temperature on herbicide efficacy and weed/crop competition. *Rept. Prairie Adapt. Res. Collab.* 1–29.

[B4] Bohm W. (1979). *Methods of Studying Root Systems.* New York, NY: Spring-Verlag.

[B5] Bright C. (1998). *Life Out of Bounds: Bioinvasion in a Borderless World.* New York, NY: W.W. Norton & Company.

[B6] Bryson C. T. Carter R. (1993). Cogongrass, *Imperata cylindrica*, in the United States. *Weed Technol.* 7 1005–1009.

[B7] Capo-chichi L. J. A. Faircloth W. H. Williamson A. G. Patterson M. G. Miller J. H. van Santen E. (2008). Invasion dynamics and genotypic diversity of Cogongrass (*Imperata cylindrica*) at the point of introduction in the Southeastern United States. *Invasive Plant Sci. Manage.* 1 133–141. 10.1614/IPSM-07-007.1

[B8] Holm L. G. Plucknett D. L. Pancho J. V. Herberger J. P. (1991). *The World(s Worst Weeds: Distribution and Biology.* Malabar, FL: Krieger Publishing.

[B9] Kimball B. A. (1983). Carbon dioxide and agricultural yield: an assemblage and analysis of 430 prior observations. *Agron. J.* 75 779–788. 10.2134/agronj1983.00021962007500050014x

[B10] Littell R. C. Milliken G. A. Stroup W. W. Wolfinger R. D. (1996). *SAS System for Mixed Models.* Cary, NC: SAS Institute, Inc.

[B11] Marble S. C. Prior S. A. Runion G. B. Torbert H. A. (2015). Control of yellow and purple nutsedge in elevated CO_2_ environments with glyphosate and halosulfuron. *Front. Plant Sci.* 6:1 10.3389/fpls.2015.00001 PMC4299438 25653664

[B12] Miller J. H. Manning S. T. Enloe S. F. (2010). “A Management Guide for Invasive Plants in Southern Forests,” in *General Technology Report SRS-*131 (Asheville, NC: U.S. Department of Agriculture Forest Service).

[B13] Mitchell R. J. Runion G. B. Prior S. A. Rogers H. H. Amthor J. S. Henning S. P. (1995). Effects of nitrogen on *Pinus palustris* foliar respiratory responses to elevated atmospheric CO_2_ concentration. *J. Exp. Bot.* 46 1561–1567.

[B14] Norby R. J. Cotrufo M. F. Ineson P. O’Neill E. G. Canadell J. G. (2001). Elevated CO_2_, litter quality, and decomposition: a synthesis. *Oecologia* 127 153–165. 10.1007/s004420000615 24577644

[B15] Pappert R. A. Hamrick J. L. Donovan L. A. (2000). Genetic variation in *Pueraria lobata* (Fabaceae), an introduced, clonal, invasive plant of the southeastern United States. *Am. J. Bot.* 87 1240–1245. 10.2307/2656716 10991894

[B16] Patterson D. T. Flint E. P. Dickens R. (1980). Effects of temperature, photoperiod, and population source on the growth of cogongrass (*Imperata cylindrica*). *Weed Sci.* 28 505–509.

[B17] Pimentel D. (2002). *Biological Invasions: Economic and Environmental Costs of Alien Plant, Animal, and Microbe Species.* Boca Raton, FL: CRC Press.

[B18] Prior S. A. Runion G. B. Rogers H. H. Torbert H. A. (2008). Effects of atmospheric CO_2_ enrichment on crop nutrient dynamics under no-till conditions. *J. Plant Nutr.* 31 758–773. 10.1080/01904160801928364

[B19] Prior S. A. Torbert H. A. Runion G. B. Rogers H. H. (2003). Implications of elevated CO_2_-induced changes in agroecosystem productivity. *J. Crop Prod.* 8 217–244. 10.1300/J144v08n01_09

[B20] Rogers H. H. Dahlman R. C. (1993). Crop responses to CO_2_ enrichment. *Vegetatio* 104/105 117–131. 10.1007/BF00048148

[B21] Rogers H. H. Heck W. W. Heagle A. S. (1983a). A field technique for the study of plant responses to elevated carbon dioxide concentrations. *Air Pollut. Control Assn. J.* 33 42–44. 10.1080/00022470.1983.10465546

[B22] Rogers H. H. Thomas J. F. Bingham G. E. (1983b). Response of agronomic and forest species to elevated atmospheric carbon dioxide. *Science* 220 428–429. 10.1126/science.220.4595.428 17831416

[B23] Rogers H. H. Runion G. B. Krupa S. V. (1994). Plant responses to atmospheric CO_2_ enrichment with emphasis on roots and the rhizosphere. *Environ. Pollut.* 83 155–189. 10.1016/0269-7491(94)90034-5 15091762

[B24] Rogers H. H. Runion G. B. Prior S. A. Torbert H. A. (1999). “Response of plants to elevated atmospheric CO_2_: root growth, mineral nutrition, and soil carbon,” in *Carbon Dioxide and Environmental Stress*, eds Luo Y. Mooney H. A. (San Diego, CA: Academic Press), 215–244.

[B25] Runion G. B. Torbert H. A. Prior S. A. Rogers H. H. (2009). “Effects of elevated atmospheric carbon dioxide on soil carbon in terrestrial ecosystems of the southeastern U.S,” in *Soil Carbon Sequestration and the Greenhouse Effect*, 2nd Edn, eds Lal R. Follett R. F. (Madison, WI: Soil Science Society of America), 233–262.

[B26] Tabor P. (1949). Cogongrass, *Imperata cylindrica* (L.) Beauv., in the southeastern United States. *Agron. J.* 41 270 10.2134/agronj1949.00021962004100060011x

[B27] Tabor P. (1952). Cogongrass in Mobile County. *Alabama. Agron. J.* 44:50 10.2134/agronj1952.00021962004400010012x

[B28] Ziska L. H. (2003). Evaluation of the growth response of six invasive species to past, present and future atmospheric carbon dioxide. *J. Exp. Bot.* 54 395–404. 10.1093/jxb/erg027 12493868

[B29] Ziska L. H. Runion G. B. (2007). “Future weed, pest, and disease problems for plants,” in *Agroecosystems in a Changing Climate*, eds Newton P. C. D. Carran R. A. Edwards G. R. Niklaus P. A. (Boca Raton, FL: CRC Press), 261–287.

[B30] Ziska L. H. Teasdale J. R. (2000). Sustained growth and increased tolerance to glyphosate observed in a C_3_ perennial weed, quackgrass (*Elytrigia repens*), grown at elevated carbon dioxide. *Aust. J. Plant Physiol.* 27 159–166.

[B31] Ziska L. H. Teasdale J. R. Bunce J. A. (1999). Future atmospheric carbon dioxide may increase tolerance to glyphosate. *Weed Sci.* 47 608–615.

